# xinguangA preliminary characterization of PI4K/PIPK alterations across solid tumors: an exploratory framework for prognostic and therapeutic stratification

**DOI:** 10.1080/15384047.2026.2692173

**Published:** 2026-07-14

**Authors:** Yaqiong Jie, Zhongyu Lu, Xiaoxuan Wang, Dongsheng Chen, Xing Zhang, Xinguang Han

**Affiliations:** a Department of Oral and Maxillofacial Surgery, The First Affiliated Hospital of Zhengzhou University, Zhengzhou, China; b State Key Laboratory of Neurology and Oncology Drug Development, Jiangsu Simcere Diagnostics Co., Ltd., Nanjing Simcere Medical Laboratory Science Co., Ltd., Nanjing, China

**Keywords:** Phosphatidylinositol 4-kinase (PI4K), phosphatidylinositol phosphate kinase (PIPK), genomic alterations, tumor immune microenvironment, solid tumors

## Abstract

**Background:**

This study aimed to systematically characterize the genomic alteration landscape of phosphatidylinositol 4-kinase (PI4K) and phosphatidylinositol phosphate kinase (PIPK) family genes in solid tumors, assess the correlation of these alterations with patient clinical outcomes and the tumor immune microenvironment, and explore their potential as novel biomarkers or therapeutic targets.

**Methods:**

A retrospective analysis was conducted on whole-exome sequencing data from a cohort of 2,144 Chinese patients encompassing 18 solid tumor types. Genomic and transcriptomic data from The Cancer Genome Atlas (TCGA) pan-cancer project were integrated to perform survival analysis, correlating genomic alterations, gene expression levels, and patient overall survival. Associations between these gene alterations and tumor mutational burden (TMB), microsatellite instability (MSI), and levels of tumor-infiltrating immune cells were also evaluated.

**Results:**

We identified PI4K/PIPK alterations in 10.5% of 2,144 patients, with PIP4K2C amplification reaching 48%. While PI4K2B amplification and overexpression are consistently associated with poor prognosis in COAD, family-wide effects were highly heterogeneous across cancers. Alterations significantly correlated with higher TMB and MSI-H, and multi-omics analysis revealed tissue-specific immune landscapes, highlighting the family's potential for risk stratification and immunotherapy.

**Conclusions:**

This study provides the first systematic delineation of the genomic alteration landscape of PI4K and PIPK families at a pan-cancer scale within a Chinese population. It identifies PI4K2B as an amplification-driven prognostic biomarker in colorectal cancer, with potential clinical value analogous to HER2. These findings illuminate the significant roles of these kinases in cancer and provide novel insights for future precision oncology strategies.

## Introduction

The precise coordination of complex cellular processes such as growth, proliferation, survival, metabolism, and motility relies on an extensive intracellular signaling network.^1^ Among the key regulators of critical cellular functions, particularly those involved in cancer initiation and progression, the phosphatidylinositol kinase family plays a central role.^2-4^ Phosphatidylinositol kinases can be broadly classified into three families: phosphatidylinositol 3-kinase (PI3K), phosphatidylinositol 4-kinase (PI4K), and phosphatidylinositol phosphate kinase (PIPK).

The PI4K family serves as the “founder” in the phosphatidylinositol metabolic pathway. They specifically catalyze the phosphorylation of the 4-hydroxy group on the inositol ring to generate PI4P. This family can be further subdivided into type II kinases, including the isoforms PI4KIIα (also known as PI4K2A) and PI4KIIβ (also known as PI4K2B), and type III kinases, including the isoforms PI4KIIIα (also known as PI4KA) and PI4KIIβ (also known as PI4KB).^5^ ^,^ ^6^ The PIPK family, also referred to as phosphatidylinositol 4-phosphate 5-kinase (PIP5K), is the primary producer of PI(4,5)P2. Type I kinases include three phosphatidylinositol 4-phosphate 5-kinases (PI4P5K, also known as PIP5K), type II kinases include three phosphatidylinositol 5-phosphate 4-kinases (PI5P4K, also known as PIP4K), and type III kinases consist of a single member, the 3-phosphatidylinositol 5-kinase (PIKfyve).^7^ ^,^ ^8^

Acting as signaling docks on the cell membrane, these kinases dynamically determine their ability to recruit and activate specific effector proteins through reversible phosphorylation of the hydroxyl groups on the inositol ring, thereby precisely regulating key cellular processes. They exhibit distinct yet collaborative biological functions, collectively constructing a complex intracellular signaling network.^9-11^ The PI3K family has garnered significant attention due to the close association of its products with diseases such as cancer and immunometabolism.^12-14^ Consequently, as core regulators of the homeostasis of these critical phospholipid molecules, dysregulation of the PI4K and PIPK families is believed to be closely linked to malignant phenotypes in tumor cells, including unlimited proliferation, metabolic reprogramming, invasion, migration, and therapy resistance.^15-17^ In contrast to PI3K, the genomic characteristics of its upstream kinases, the PI4K and PIPK families, in solid tumors, how these alterations affect patient clinical outcomes, and whether they can serve as independent drivers or potential therapeutic targets remain poorly understood.

This study conducts a comprehensive genomic analysis of the PIPK and PI4K families, revealing their specific mutation patterns in solid tumors. Additionally, we assess the correlation between their alterations and clinical outcomes through survival analysis. By integrating the assessment of immune cell infiltration and its relationship with these genomic alterations, we aim to uncover their potential roles of these kinases in tumorigenesis and their prospects as biomarkers or therapeutic targets within the tumor microenvironment.

## Results

### Pan-cancer alteration profile

**Figure 1.**
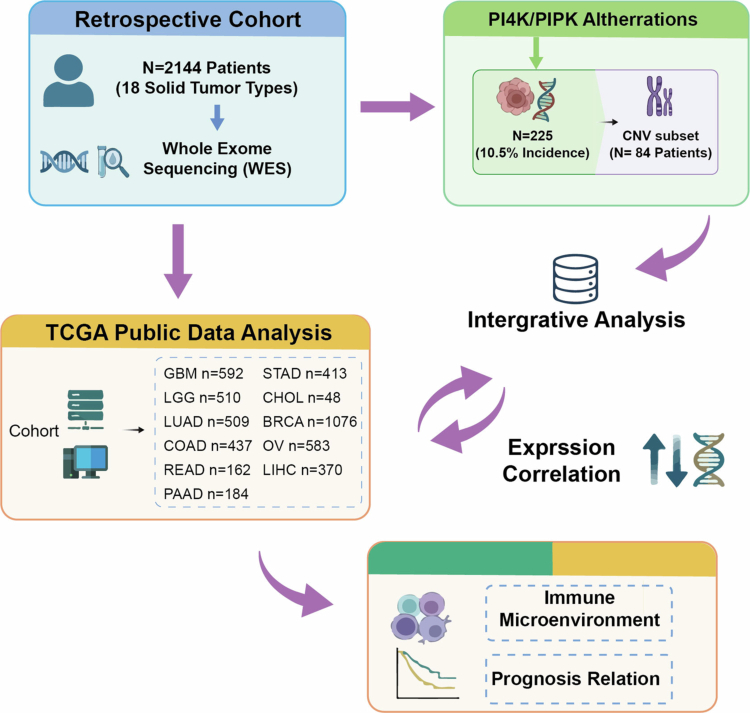
Schematic overview of the analytical pipeline for PI4K/PIPK alterations.

In this retrospective study, we analyzed whole-exome sequencing data from tumor samples of 2,144 patients, encompassing 18 solid tumor types. Among these patients, 53.5% (*n* = 1,148) were male, and 16.2% (*n* = 347) were classified as stage IV at diagnosis. The detailed workflow for patient screening and genomic analysis is illustrated in Figure 1. We identified genomic alterations in the PI4K and PIPK families in 225 patients, corresponding to an overall frequency of 10.5%. Among these 225 patients, glioma (28%) was the most common tumor type, followed by lung cancer (16.9%), colorectal cancer (13.3%), gastric cancer (7.6%), and biliary tract cancer (4.9%) (Table 1).

**Table 1.** Baseline characteristics by PI4K and PIPK family genomic alteration status.

Character	Mutated (*n* = 225)	Wild-type (*n* = 1919)
Median age, years (range)	54 (3, 84)	52 (0, 84)
**Sex, *n* (%)**		
Male	120 (53.3)	1,028 (53.6)
Female	105 (46.7)	891 (46.4)
**Stage, *n* (%)**		
I	8 (3.6)	106 (5.5)
II	17 (7.5)	216 (11.3)
III	27 (12)	217 (11.3)
IV	33 (14.7)	315 (16.4)
Unknown	140 (62.2)	1,065 (55.5)
**Tumor type, *n* (%)**		
Glioma	63 (28)	586 (30.5)
Lung cancer	38 (16.9)	394 (20.5)
Colorectal cancer	30 (13.3)	187 (9.7)
Pancreatic cancer	9 (4)	98 (5.1)
Gastric cancer	17 (7.6)	83 (4.3)
Medulloblastoma	1 (0.4)	80 (4.2)
Biliary cancer	11 (4.9)	69 (3.6)
Breast cancer	10 (4.4)	68 (3.5)
Ovarian cancer	8 (3.6)	69 (3.6)
Liver cancer	9 (4)	61 (3.2)

**Figure 2.**
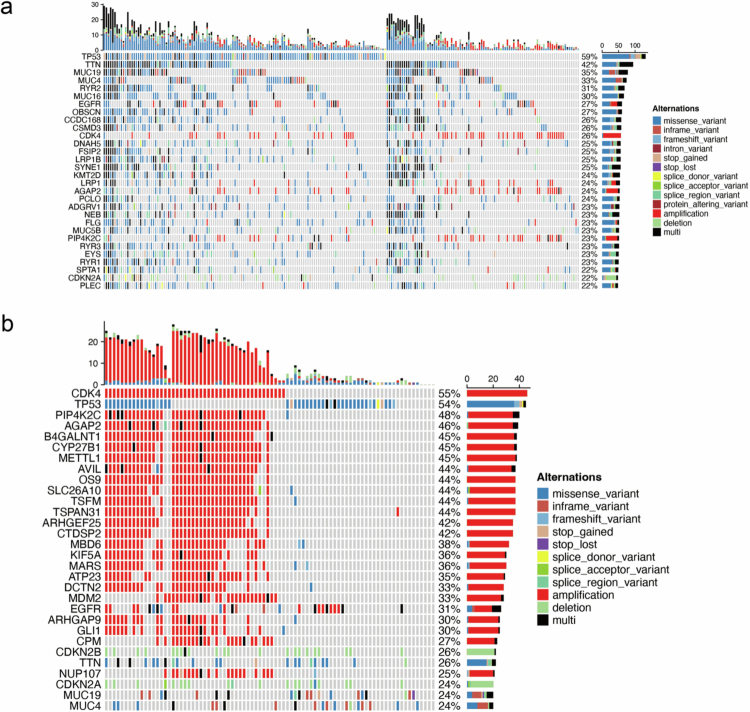
Mutation profiles of patients with alterations in PI4K and PIPK family genes. a, Mutational landscape of 225 patients harboring variants in PI4K and PIPK family genes. b, Copy number alteration profile of 84 patients with copy number variations in PI4K and PIPK family genes.

Subsequently, we generated a mutational landscape for the 225 patients harboring PI4K and PIPK family alterations (Figure 2a). This analysis revealed that TP53, TTN, MUC19, MUC4, and RYR2 were the most frequently co-occurring alterations. Furthermore, the mutational landscape for the 84 patients with PI4K and PIPK family copy number variations (CNVs) was also depicted (Figure 2b). Here, CDK4, TP53, AGAP2, B4GALNT1, and CUP27B1 emerged as the most common co-alterations. Interestingly, these co-mutations were also predominantly amplifications. Notably, we observed PIP4K2C gene amplification in 48% of these patients. Single-nucleotide polymorphisms (SNPs) were the most prevalent alteration type overall, followed by amplifications and deletions.

### Tumor type-specific alteration patterns

**Figure 3.**
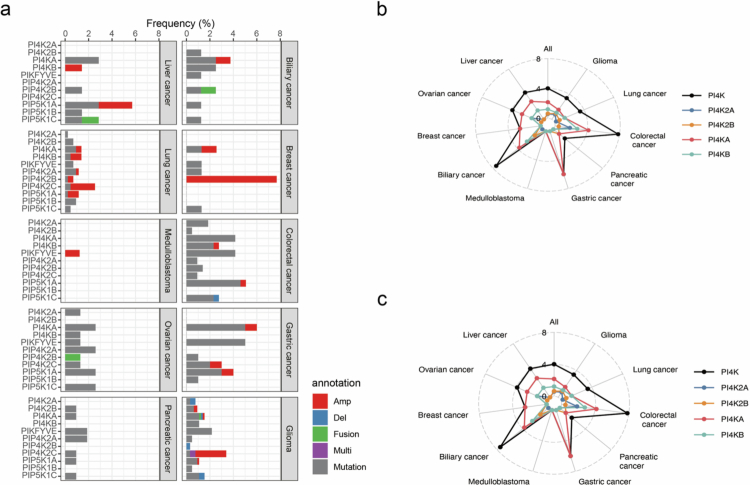
The alteration landscape of PI4K and PIPK family genes across different cancer types. a, Mutation types of individual PI4K and PIPK family genes in various cancers. The bar plot shows the frequency of alterations, categorized as Amplification (Amp), Deletion (Del), Fusion, Multi-mutation (Multi), or Mutation. b-c, Alteration frequencies of PI4K (b) and PIPK (c) family genes across cancer types.

Although SNPs were the most common alteration type, the distribution of PI4K and PIPK family mutations varied across different solid tumor types (Figure 3a). For instance, in ovarian and pancreatic cancers, almost all genes in these families were presented as SNPs. In glioma, the mutation spectrum was more complex, exhibiting a combination of amplifications, deletions, fusions, and SNPs. In lung cancer, amplifications were observed in multiple PI4K and PIPK family genes. Within the PI4K family, PI4KA was the most frequently altered gene, whereas PIP4K2C was the most commonly altered in the PIPK family (Figure 3b-c). Colorectal cancer showed a higher propensity for PI4K family alterations, while gastric cancer was more associated with PIPK family variants.

### Pan-cancer prognostic associations of genomic and expression alterations

**Figure 4.**
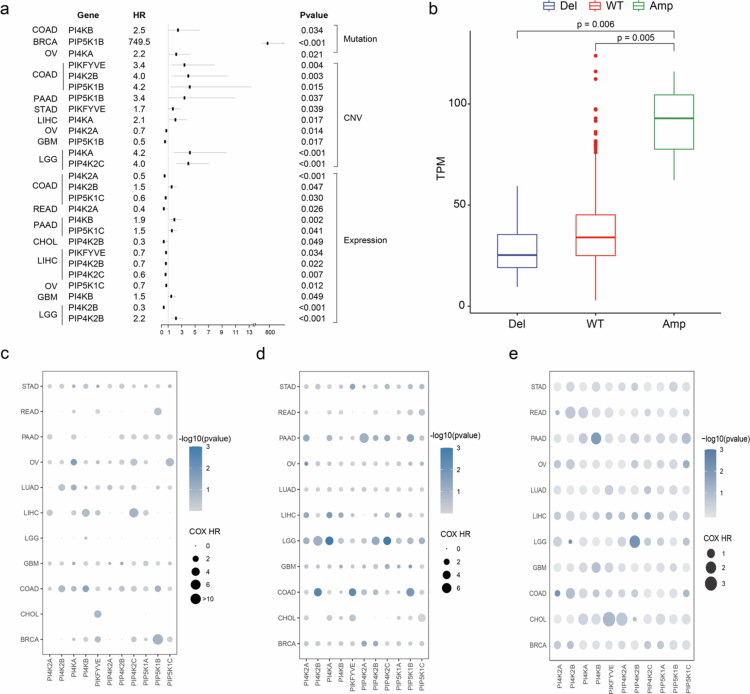
Prognostic associations of genetic and expression alterations in PI4K and PIPK family genes across cancer types. a, Forest plot showing the impact on prognosis (Hazard Ratio, HR) of specific mutations and CNVs in individual PI4K and PIPK family genes across different cancer types. *P*-values indicate statistical significance. b, Analysis of the correlation between gene copy number alterations and mRNA expression levels. The plot displays the association between CNV status (Deletion, Wild-Type, Amplification) and gene expression (TPM) for representative genes. c–e, Bubble plots illustrating the prognostic associations across cancer types. Prognostic impact of mutations (b), CNVs (c) and gene expression levels (d) in PI4K and PIPK family genes. The bubble plot displays the -log10(*P*-value) on the Y-axis against the Hazard Ratio (HR) on the X-axis. Bubble size and color intensity often represent effect size or statistical power.

To systematically evaluate the prognostic value of PI4K and PIPK family gene alterations in cancer, we integrated an analysis of their mutations, CNV, and expression data from a The Cancer Genome Atlas (TCGA) pan-cancer cohort (with comprehensive cohort profiles detailed in Supplementary Table 1-4) with patient overall survival (Figure 4a). Our analysis revealed that this gene family exhibits multi-layered and heterogeneous prognostic associations across various cancer types. We identified PI4K2B as a gene of particular interest in colon adenocarcinoma (COAD), where its copy number amplification was associated with poor prognosis. To investigate the potential mechanism underlying this association, we assessed the relationship between copy number and mRNA expression of PI4K2B specifically in the COAD cohort. This analysis confirmed a strong positive correlation, wherein gene amplification was significantly associated with elevated transcript levels (Figure 4b). This suggests that the poor prognosis linked to PI4K2B amplification may be driven by its increased expression.

This mechanistic link was further solidified by survival analysis at the gene expression level, which demonstrated that high PI4K2B expression was itself significantly associated with poorer survival in COAD (HR > 1, *p* < 0.05) (Figure 4e). Together, these findings at the genomic and transcriptomic levels suggest that PI4K2B serves as a potential prognostic risk factor in COAD. Correlation analysis further demonstrated that PI4K2B expression was significantly associated with increased infiltration of CD8+ T cells within the COAD microenvironment (Figure 6c). Given that dense T-cell infiltration is a prominent feature of MSI-High (MSI-H) colorectal tumors, a stratified analysis based on microsatellite status was performed to evaluate potential confounding effects. The positive correlation between PI4K2B expression and CD8+ T-cell recruitment was found to be exclusively maintained within the MSS subgroup (R = 0.26, *p* < 0.05), whereas no statistically significant association was observed in the MSI-H population (R = 0.0019, *p* = 0.99) (Supplementary Figure 1). However, the relatively low alteration frequency of PI4K2B in both the present research cohort and the TCGA cohort may limit the broader generalizability of this finding.

**Figure 5.**
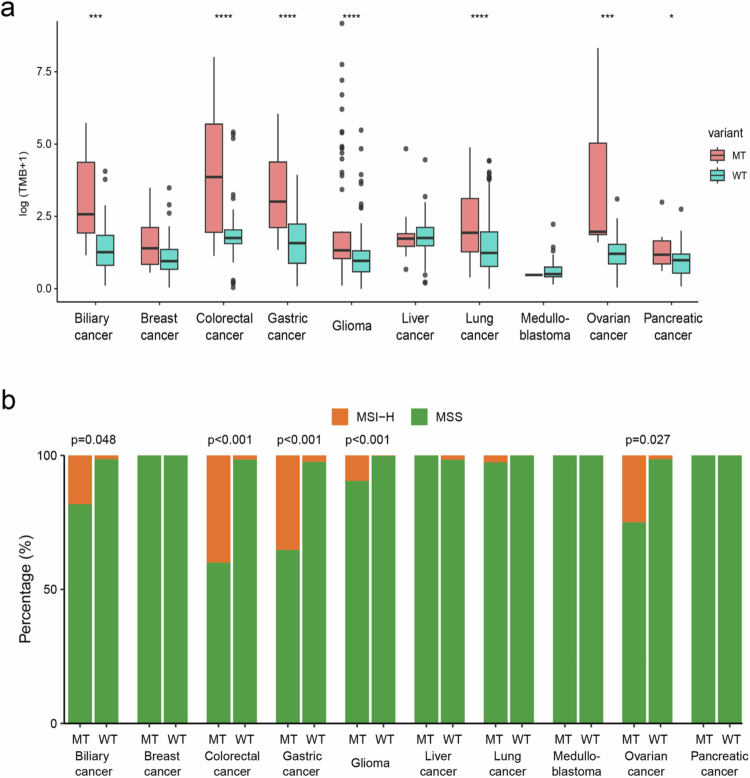
TMB and MSI status (MSI-H vs. MSS) according to PI4K/PIPK alterations across cancer types. a, Box plots comparing TMB between tumors with mutant (MT) and wild-type (WT) PI4K/PIPK genes across different cancer types. b, Association between PI4K/PIPK gene mutations and MSI status (MSI-H vs. MSS) in specific cancer types, with corresponding *p*-values from statistical tests.

Beyond this focused finding, our analysis revealed additional associations (Figure 4c–e). At the mutation level, mutations in genes such as PI4KB in COAD and PIP5K1B in breast invasive carcinoma (BRCA) were also linked to poorer survival (HR > 1, *p* < 0.05). Similarly, amplification of PIKFYVE was indicative of poor prognosis in both stomach adenocarcinoma (STAD) and COAD, suggesting its potential role as a risk factor in gastrointestinal cancers. Conversely, CNVs of PI4K2A in ovarian serous cystadenocarcinoma (OV) were associated with prolonged survival (HR < 1, *p* < 0.05), hinting at potential tissue-specific functions.

### Correlations with immunotherapeutic biomarkers and immune features

Next, we investigated the correlation between PI4K and PIPK family mutations and the immune checkpoint inhibitor biomarkers, tumor mutational burden (TMB) and microsatellite instability (MSI), across different tumors. The results demonstrated that the mutation group exhibited a significantly higher TMB (Figure 5a), with statistical significance observed in biliary tract cancer, colorectal cancer, gastric cancer, glioma, lung cancer, ovarian cancer, and pancreatic cancer. Furthermore, the mutation group was also more prone to MSI-H status (Figure 5b), reaching statistical significance in biliary tract cancer, colorectal cancer, gastric cancer, glioma, and ovarian cancer.

**Figure 6.**
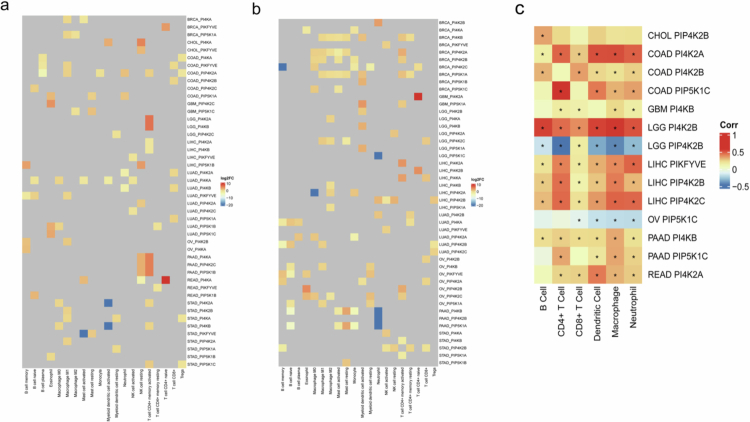
Association of PI4K and PIPK family gene alterations with immune cell infiltration. a, mutations, b, CNVs, and c, gene expression. Correlation coefficients are color‑coded.

To systematically assess how the PI4K/PIPK family genes regulate the tumor immune microenvironment, we analyzed their correlations with multiple immune cell infiltration levels at three levels (Figure 6a–c): mutation, CNV, and gene expression. Our results reveal that these genes shape the immune landscape in a highly cancer‑type‑specific manner and exhibit functional heterogeneity across different omics layers.

In LIHC, we observed a consistent immune‑activating signal across multi‑omics data. Gene expression analysis showed that PIP4K2C and PIP4K2B levels were significantly positively correlated with infiltration of CD8+ T cells, macrophages, and neutrophils. In line with this, copy number gains of PI4KA and PI4K2B, as well as mutations in PIP5K1B, were markedly associated with recruitment of T cell subsets. These findings suggest that both genetic alterations and upregulation of these genes contribute to an immune-enriched tumor microenvironment in LIHC, offering preliminary insights into their potential roles in modulating immunotherapy response.

Unlike LIHC, LGG displayed a complex molecular switch effect. At the expression level, PIP4K2B was significantly negatively correlated with nearly all immune cell types analyzed. At the mutation level, however, mutations in PI4K2A and PI4KB showed an extremely strong positive association with activated CD4+ memory T cells. Analysis of PAAD revealed a polarity difference between CNV and mutation in their immune correlations. CNV data indicated that variants of PI4KB and PIP4K2B were extremely negatively associated with neutrophil and monocyte infiltration. In contrast, mutations in PI4KA and PIP5K1B were significantly linked to increased infiltration of activated CD4+ memory T cells. In addition, the regulation of specific immune subsets by this family displayed clear tissue preference. For example, in breast cancer, genes such as PIP4K2A mainly influenced the M1/M2 polarization trend of macrophages; in rectal cancer (READ), mutations in PI4KA were tightly correlated with strong enrichment of naïve CD4+ T cells.

Taken together, members of the PI4K and PIPK families fine‑tune immune cell recruitment and functional status across different cancer types through interactions among multiple dimensions, including gene expression, copy number variation, and somatic mutation. These findings provide important insights into the complex role of the phosphoinositide metabolism pathway in tumor immunity and highlight its potential value as a biomarker or therapeutic target for immunotherapy.

## Discussion

This study represents the first systematic delineation of the alteration landscape of PI4K and PIPK family genes within a Chinese population at a pan-cancer scale. It further provides an in-depth exploration of their associations with patient prognosis and the tumor immune microenvironment. Our findings illuminate the significant roles these phosphoinositide kinases play in cancer and offer novel insights into their potential applications in precision medicine.

Through systematic genomic analysis, this study reveals that the relatively understudied PI4K and PIPK members of the phosphoinositide kinase family harbor non-negligible alterations across multiple solid tumors. The overall alteration frequency of 10.5%, particularly their high prevalence in glioma, lung cancer, and gastrointestinal cancers, strongly suggests that this gene family plays a role beyond that of a mere bystander in tumorigenesis and progression. The remarkably high amplification frequency of the PIP4K2C gene (48%) in the CNV cohort is a critical finding. Although the role of PIP4K2C in cancer remains insufficiently explored, its product, PI5P4K, has been shown to indirectly influence the p53 signaling pathway and cellular stress response by synthesizing PI(4,5)P2 and consuming PI5P.^18^ ^,^ ^19^ Its high-frequency amplification might, by increasing gene dosage, disrupt the balance of lipid second messengers, thereby conferring growth or survival advantages to cancer cells. As a PI5P kinase, the overexpression of PIP4K2C is expected to reconfigure the phosphoinositide landscape by depleting the substrate PI5P and promoting the accumulation of PI(4,5)P2. The enrichment of PI(4,5)P2 provides an abundant substrate pool for the PI3K/AKT axis, thereby fueling cell survival and proliferation. Conversely, the exhaustion of PI5P—a stress-responsive signaling molecule—may dampen cellular stress responses and suppress autophagy,^20^ potentially conferring a survival advantage to malignant cells. Notably, the PIP4K family has emerged as a compelling synthetic lethal vulnerability in p53-deficient tumors. Our observation of frequent PIP4K2C amplification may thus represent a non-random, compensatory adaptation to p53 inactivation. In the absence of functional p53, tumor cells may become hyper-dependent on PIP4K activity to maintain PI(4,5)P2 levels and sustain indispensable AKT signaling. We hypothesize that this gene dosage effect serves to reconstruct lipid messenger homeostasis, thereby supporting aggressive progression under genomic instability. These findings highlight PIP4K2C not only as a promising prognostic indicator but also as a strategic target for synthetic lethal interventions in p53-mutant cancers.^21^ Furthermore, the co-mutation landscape reveals the frequent co-occurrence of these kinase alterations with established oncogenes such as TP53 and CDK4. The specific co-occurrence with CDK4 amplification suggests a potential functional synergy between PI4K/PIPK signaling and cell cycle regulation pathways, collectively driving proliferative signals.^22^ ^,^ ^23^

In the prognostic analysis, the most striking finding was the consistent and independent prognostic value of PI4K2B in COAD. Our data demonstrated that both copy number amplification of PI4K2B and its upregulated mRNA expression were significantly associated with poorer overall survival in patients. This strongly indicates that the high expression of PI4K2B is likely driven by its gene amplification events. This gene dosage effect closely mirrors the classic paradigm observed with ERBB2 gene amplification, which leads to HER2 protein overexpression and drives malignant progression in breast cancer.^24-26^

Notably, members of the PI4K II family show divergent prognostic effects depending on the cancer type, highlighting a high degree of context dependency. While PI4K2B acts as a significant risk factor in colorectal cancer, PI4K2A is associated with significantly longer survival in ovarian cancer. We hypothesize that these functional dichotomies stem from tissue-specific protein interactomes and the organ microenvironment.^27^ ^,^ ^28^ In colorectal cancer, PI4K2B may be recruited to signaling complexes on the endosomal surface to promote growth; by creating a localized pool of PI4P, it provides a platform for the sustained activation of oncogenic pathways such as KRAS.^29^ In contrast, in the context of ovarian cancer, the family appears more involved in endosomal trafficking or autophagy to maintain genomic stability, potentially inhibiting malignancy by facilitating the lysosomal degradation of damaged organelles.^30^ Furthermore, the distinct metabolic needs of various organs may reshape how these kinases function by altering the flux of the phosphoinositide cycle.

Despite this pan-cancer heterogeneity, the oncogenic involvement of PI4K2B in the colorectal landscape suggests its potential clinical relevance for a specific patient subset. In clinical molecular testing, HER2 status is determined by combining immunohistochemistry (for protein expression) and FISH (for gene amplification).^31^ Similarly, our study suggests that PI4K2B may possess analogous biomarker potential in colorectal cancer. Future clinical practice could consider combining the detection of PI4K2B CNV status and its expression level to more accurately identify a subgroup of COAD patients with highly aggressive clinical features. This finding not only provides a new molecular indicator for risk stratification in colorectal cancer but also establishes a theoretical foundation for exploring targeted therapies against PI4K2B.

In the era of cancer immunotherapy, identifying patient populations likely to benefit from immune checkpoint inhibitors (ICIs) is paramount.^32^ A key finding of our study is that tumors harboring PI4K/PIPK family alterations exhibited significantly higher TMB and MSI-H status. It is well-established that elevated TMB and MSI-H can generate an abundance of neoantigens, thereby enhancing tumor immunogenicity and serving as validated biomarkers for predicting response to immunotherapy.^33-36^ Notably, our data revealed that across different cancer types, all cases where the alteration-positive group showed a significantly higher frequency of MSI-H status also exhibited significantly higher TMB. However, the converse was not always true, as some tumor types demonstrated a significant increase in TMB without a corresponding shift in MSI-H status. This observation underscores that TMB, as a continuous quantitative metric, captures subtle fluctuations in genomic mutational burden with higher resolution than the binary MSI-H status. Consequently, TMB may offer a more sensitive indicator for identifying patients with PI4K/PIPK family alterations who could potentially benefit from ICIs,^37^ especially in cancer types where MSI-H events are rare. Taken together, while MSI-H remains a robust categorical biomarker, integrating TMB as a complementary dimension may refine patient selection in future studies.

Furthermore, our correlation analysis substantiates a complex network linking these genes to immune cell infiltration within the tumor microenvironment. Specifically in COAD, we observed that PI4K2B expression is significantly associated with increased infiltration of CD8+ T cells, an association that our stratified data shows is uniquely sustained within the MSS subset. Although a high density of infiltrating lymphocytes is traditionally linked to an immune-enriched microenvironment,^38^ this enhanced infiltration does not invariably translate into an effective anti-tumor response or improved survival. Our data indicate that despite higher infiltration, these patients exhibit poorer overall survival, suggesting a potential decoupling of immune cell recruitment from functional potency. In the context of chronic tumor progression, CD8+ T cells may exist in a hyporesponsive or altered functional state, characterized by the sustained expression of inhibitory receptors such as PD-1, LAG-3, and TIM-3, alongside the diminished secretion of effector cytokines.^39^ Therefore, PI4K2B-mediated alterations may drive an immune dysfunctional landscape, where high cellular infiltration is offset by suppressive signaling, ultimately favoring malignant progression rather than effective tumor clearance. Although these specific T cell exhaustion markers were not directly validated in our study, evaluating their expression patterns remains a critical objective for future research to precisely define this dysfunctional immune landscape, thereby highlighting the importance of evaluating immune cell functionality beyond mere numerical presence when defining the prognostic impact of PI4K and PIPK family members.

Several limitations of this study should be acknowledged. First, although we characterized the mutation and CNV profiles of PI4K/PIPK family genes in a Chinese cohort of 2,144 patients, the retrospective nature of the collection and the lack of long-term survival follow-up precluded a direct assessment of the prognostic impact of these genetic alterations. This necessitated the integration of survival data from the TCGA cohort. Second, inherent differences exist between the Chinese and TCGA cohorts regarding ethnic composition, sample sources, and sequencing platforms. While our supplementary analysis showed no significant differences in mutation frequencies for the majority of genes between Asians and non-Asians within the TCGA cohort, a small subset of genes such as PI4K2A did exhibit divergent alteration patterns. Consequently, the statistical power to resolve race-specific variations is limited, and the risk of potential bias associated with cross-dataset integration cannot be entirely eliminated. Third, the functional associations between genetic alterations and the immune microenvironment reported here have not been validated through in vitro or in vivo experiments. Furthermore, as all findings were derived from retrospective data analysis, they should be considered exploratory. Consequently, our results remain preliminary and warrant further validation in larger, multi-center, prospective cohorts, alongside functional studies to elucidate the underlying molecular mechanisms.

## Conclusion

In summary, this study establishes multidimensional associations between genetic alterations in PI4K/PIPK family genes and both poor prognosis and immune microenvironment features across multiple cancer types. We specifically identified PI4K2B as an amplification-driven prognostic biomarker in colorectal cancer, demonstrating localized clinical relevance that warrants further validation. Furthermore, the high TMB/MSI characteristics associated with mutations in this gene family provide a strong rationale for considering immunotherapy in these patients. Future research should focus on elucidating the precise molecular mechanisms by which these kinases regulate immune responses and explore their potential as novel targets for combination strategies involving targeted therapy and immunotherapy, ultimately advancing the field of precision oncology.

## Materials and methods

### Patient cohort

This retrospective study enrolled a total of 2,421 patients with more than 20 cancer types who underwent whole exome sequencing (WES) between April 2020 and October 2024. Collected clinical data included age, sex, tumor stage, and tumor type. Genomic and transcriptomic data, along with clinical information from the TCGA Project, were obtained via the UCSC Xena platform.^40^ The study was conducted in accordance with the Declaration of Helsinki and received approval from the Ethics Committee of Nanjing Simcere Medical Laboratory (No. NSML-IRB-202406-MS18), and written informed consent for participation was obtained from all participants.

### Library preparation and NGS sequencing

Genomic DNA was extracted from patient samples using the DNeasy Tissue Kit. Libraries were constructed with the KAPA Library Preparation Kit, and library concentration was assessed using the Invitrogen Qubit 4.0. Sequencing was performed on either an Illumina NextSeq 550 or NovaSeq 6000 system, with an average depth of 500×. All WES procedures were conducted by Simcere Diagnostics Co., Ltd. (Nanjing, China), a facility certified by the Clinical Laboratory Improvement Amendments (CLIA), the College of American Pathologists (CAP), and ISO15189.

### Variant calling and bioinformatics analysis

Adapter sequences and low-quality bases were trimmed using fastp (v0.19.5),^41^ and reads shorter than 50 bp were discarded. Clean reads were aligned to the hg19 human reference genome (NCBI GRCh37; hg19)^42^ with BWA (v0.7.17)^43^ to generate BAM files. PCR duplicates were marked and removed using Sambamba (v0.6.8).^44^ To improve alignment accuracy for insertions/deletions (INDELs) and complex mutations, local realignment was performed with Abra2 (v2.21).^45^ Variants including single-nucleotide variants (SNVs), INDELs, and complex mutations were called using VarDict (v1.7.0)^46^ and annotated with SnpEff (v4.3)^47^ and ANNOVAR^48^ incorporating multiple databases (COSMIC v98, gnomAD v2, ExAC, 1000 Genomes, and dbSNP). Tumor-derived variants with fewer than 5 supporting reads or a mutation variant allele frequency (VAF) below 1% were excluded. Copy number variants (CNVs) were identified using CNVkit (dx1.1), and fusion events were called with FACTERA (v1.4.4). MSI-H was defined as a cutoff of 0.15. TMB was calculated as the total number of SNVs and indels per megabase in the coding region.

### Statistical analysis

All analyzes were performed in R (version 4.3.2). Genomic landscapes were visualized using the ComplexHeatmap package (v2.18.0). Radar plots were generated with ggradar (v0.2), bar plots and bubble plots with ggplot2 (v3.5.2), and forest plots with forestplot (v3.1.7). Cox proportional hazards regression analysis was performed using the log-rank test via the Survival (v3.5.7) and Survminer (v0.4.9) packages. Group differences in TMB, MSI, and CNV were evaluated using the Wilcoxon test, while categorical variables were compared using the chi-square test or Fisher's exact test, as appropriate. A *p*-value < 0.05 was considered statistically significant.

## Supplementary Material

Supplementary Table 3.doc Supplementary Table 3.doc

Supplementary Table 1.doc Supplementary Table 1.doc

Supplementary figure 1.jpg Supplementary figure 1.jpg

Supplementary Table 2.doc Supplementary Table 2.doc

Supplementary Table 4.doc Supplementary Table 4.doc

Supplementary Material Supplementary Material

## Data Availability

Researchers who request access to raw and analyzed data should send an email to the corresponding author XG. Han to clarify the research purpose. Data are available for approved eligible applications and investigators, after signing a data access agreement.
