# Supplementary material for: xinguangA preliminary characterization of PI4K/PIPK alterations across solid tumors: an exploratory framework for prognostic and therapeutic stratification
Source: Cancer Biol Ther. 2026 Jul 14;27(1):2692173. doi: 10.1080/15384047.2026.2692173 (PMC13371475; doi:10.1080/15384047.2026.2692173)
Supplement: Supplementary Table 3.doc [file KCBT_A_2692173_SM8508.doc]

**Supplementary Table 3** Patient counts of PI4K/PIPK variants by race in the TCGA cohort

| **cancer** | **Patients** | | | **PI4K** | | **PI4K2A** | | **PI4K2B** | | **PI4KA** | | **PI4KB** | |
| --- | --- | --- | --- | --- | --- | --- | --- | --- | --- | --- | --- | --- | --- |
|  | **all** | **asian** | **non-asian** | **asian** | **non-asian** | **asian** | **non-asian** | **asian** | **non-asian** | **asian** | **non-asian** | **asian** | **non-asian** |
| BRCA | 1002 | 61 | 941 | 3 | 25 | 1 | 2 | 0 | 3 | 1 | 16 | 1 | 10 |
| CHOL | 50 | 3 | 47 | 0 | 0 | 0 | 0 | 0 | 0 | 0 | 0 | 0 | 0 |
| COAD | 285 | 11 | 274 | 3 | 29 | 2 | 2 | 1 | 5 | 1 | 16 | 1 | 12 |
| READ | 89 | 1 | 88 | 0 | 7 | 0 | 0 | 0 | 1 | 0 | 6 | 0 | 0 |
| GBM | 571 | 13 | 558 | 0 | 14 | 0 | 3 | 0 | 3 | 0 | 8 | 0 | 5 |
| LGG | 505 | 8 | 497 | 0 | 7 | 0 | 0 | 0 | 1 | 0 | 5 | 0 | 3 |
| LIHC | 367 | 161 | 206 | 4 | 7 | 0 | 2 | 0 | 0 | 3 | 5 | 2 | 0 |
| LUAD | 454 | 8 | 446 | 0 | 31 | 0 | 2 | 0 | 1 | 0 | 16 | 0 | 13 |
| OV | 555 | 20 | 535 | 0 | 27 | 0 | 1 | 0 | 5 | 0 | 11 | 0 | 12 |
| PAAD | 180 | 11 | 169 | 0 | 2 | 0 | 2 | 0 | 0 | 0 | 1 | 0 | 1 |
| STAD | 381 | 89 | 292 | 0 | 26 | 0 | 4 | 0 | 5 | 0 | 10 | 0 | 15 |
| **cancer** | **Patients** | | | **PIPK** | | **PIP5K1A** | | **PIP5K1B** | | **PIP5K1C** | | **PIP4K2A** | |
|  | **all** | **asian** | **non-asian** | **asian** | **non-asian** | **asian** | **non-asian** | **asian** | **non-asian** | **asian** | **non-asian** | **asian** | **non-asian** |
| BRCA | 1002 | 61 | 941 | 1 | 39 | 1 | 10 | 0 | 4 | 0 | 3 | 0 | 3 |
| CHOL | 50 | 3 | 47 | 0 | 3 | 0 | 1 | 0 | 0 | 0 | 0 | 0 | 0 |
| COAD | 285 | 11 | 274 | 3 | 37 | 1 | 1 | 0 | 4 | 1 | 12 | 0 | 10 |
| READ | 89 | 1 | 88 | 0 | 7 | 0 | 1 | 0 | 2 | 0 | 0 | 0 | 3 |
| GBM | 571 | 13 | 558 | 0 | 33 | 0 | 5 | 0 | 5 | 0 | 4 | 0 | 5 |
| LGG | 505 | 8 | 497 | 0 | 9 | 0 | 1 | 0 | 1 | 0 | 1 | 0 | 1 |
| LIHC | 367 | 161 | 206 | 9 | 10 | 1 | 1 | 2 | 0 | 0 | 1 | 0 | 1 |
| LUAD | 454 | 8 | 446 | 0 | 38 | 0 | 3 | 0 | 7 | 0 | 2 | 0 | 4 |
| OV | 555 | 20 | 535 | 1 | 35 | 1 | 5 | 0 | 2 | 0 | 2 | 0 | 3 |
| PAAD | 180 | 11 | 169 | 1 | 1 | 0 | 1 | 0 | 1 | 0 | 1 | 1 | 0 |
| STAD | 381 | 89 | 292 | 12 | 28 | 1 | 2 | 0 | 1 | 4 | 10 | 0 | 7 |
| **cancer** | **Patients** | | | **PIP4K2B** | | **PIP4K2C** | | **PIKFYVE** | |  |  |  |  |
|  | **all** | **asian** | **non-asian** | **asian** | **non-asian** | **asian** | **non-asian** | **asian** | **non-asian** |  |  |  |  |
| BRCA | 1002 | 61 | 941 | 0 | 2 | 0 | 11 | 0 | 18 |  |  |  |  |
| CHOL | 50 | 3 | 47 | 0 | 0 | 0 | 0 | 0 | 2 |  |  |  |  |
| COAD | 285 | 11 | 274 | 0 | 5 | 0 | 1 | 1 | 37 |  |  |  |  |
| READ | 89 | 1 | 88 | 0 | 2 | 0 | 0 | 0 | 2 |  |  |  |  |
| GBM | 571 | 13 | 558 | 0 | 7 | 0 | 3 | 0 | 15 |  |  |  |  |
| LGG | 505 | 8 | 497 | 0 | 1 | 0 | 2 | 0 | 4 |  |  |  |  |
| LIHC | 367 | 161 | 206 | 0 | 1 | 1 | 1 | 5 | 5 |  |  |  |  |
| LUAD | 454 | 8 | 446 | 0 | 2 | 0 | 8 | 0 | 17 |  |  |  |  |
| OV | 555 | 20 | 535 | 0 | 4 | 0 | 2 | 0 | 19 |  |  |  |  |
| PAAD | 180 | 11 | 169 | 0 | 1 | 0 | 1 | 0 | 0 |  |  |  |  |
| STAD | 381 | 89 | 292 | 3 | 4 | 2 | 1 | 3 | 15 |  |  |  |  |
