# Supplementary material for: xinguangA preliminary characterization of PI4K/PIPK alterations across solid tumors: an exploratory framework for prognostic and therapeutic stratification
Source: Cancer Biol Ther. 2026 Jul 14;27(1):2692173. doi: 10.1080/15384047.2026.2692173 (PMC13371475; doi:10.1080/15384047.2026.2692173)
Supplement: Supplementary Table 1.doc [file KCBT_A_2692173_SM8504.doc]

**Supplementary Table 1** Number of patients with PI4K/PIPK variants in the TCGA cohort

| **Cancer** | **Patients** | **Num** | **PI4K** | **PI4K2A** | | | | | | **PI4K2B** | | | | | |
| --- | --- | --- | --- | --- | --- | --- | --- | --- | --- | --- | --- | --- | --- | --- | --- |
|  |  |  |  | **All** | **Amp** | **Del** | **Mutation** | **Fusion** | **Multi** | **All** | **Amp** | **Del** | **Mutation** | **Fusion** | **Multi** |
| BRCA | 1076 | 239 | 132 | 9 | 1 | 2 | 3 | 2 | 1 | 8 | 2 | 3 | 3 | 0 | 0 |
| LUAD | 509 | 84 | 53 | 5 | 0 | 2 | 3 | 0 | 0 | 4 | 2 | 0 | 2 | 0 | 0 |
| COAD | 437 | 43 | 21 | 5 | 0 | 3 | 2 | 0 | 0 | 2 | 0 | 0 | 2 | 0 | 0 |
| READ | 162 | 23 | 14 | 0 | 0 | 0 | 0 | 0 | 0 | 2 | 0 | 0 | 2 | 0 | 0 |
| GBM | 592 | 70 | 13 | 0 | 1 | 0 | 2 | 0 | 0 | 4 | 0 | 0 | 4 | 0 | 0 |
| LGG | 510 | 57 | 12 | 0 | 0 | 0 | 0 | 0 | 0 | 2 | 1 | 0 | 1 | 0 | 0 |
| LIHC | 370 | 74 | 51 | 3 | 1 | 0 | 2 | 0 | 0 | 1 | 1 | 0 | 0 | 0 | 0 |
| PAAD | 184 | 20 | 8 | 1 | 0 | 0 | 1 | 0 | 0 | 0 | 0 | 0 | 0 | 0 | 0 |
| CHOL | 48 | 10 | 5 | 0 | 0 | 0 | 0 | 0 | 0 | 0 | 0 | 0 | 0 | 0 | 0 |
| STAD | 413 | 118 | 58 | 8 | 0 | 2 | 6 | 0 | 0 | 6 | 0 | 2 | 4 | 0 | 0 |
| OV | 583 | 107 | 58 | 4 | 2 | 2 | 0 | 0 | 0 | 8 | 3 | 1 | 4 | 0 | 0 |
| **Cancer** | **Patients** | **Num** | **PI4K** | **PI4KA** | | | | | | **PI4KB** | | | | | |
|  |  |  |  | **All** | **Amp** | **Del** | **Mutation** | **Fusion** | **Multi** | **All** | **Amp** | **Del** | **Mutation** | **Fusion** | **Multi** |
| BRCA | 1076 | 239 | 132 | 23 | 7 | 2 | 13 | 1 | 0 | 94 | 89 | 0 | 5 | 0 | 0 |
| LUAD | 509 | 84 | 53 | 12 | 3 | 1 | 8 | 0 | 0 | 38 | 34 | 0 | 3 | 0 | 1 |
| COAD | 437 | 43 | 21 | 14 | 0 | 0 | 14 | 0 | 0 | 3 | 1 | 0 | 2 | 0 | 0 |
| READ | 162 | 23 | 14 | 10 | 1 | 0 | 9 | 0 | 0 | 3 | 3 | 0 | 0 | 0 | 0 |
| GBM | 592 | 70 | 13 | 4 | 2 | 1 | 1 | 0 | 0 | 3 | 2 | 0 | 1 | 0 | 0 |
| LGG | 510 | 57 | 12 | 5 | 1 | 0 | 3 | 1 | 0 | 5 | 3 | 0 | 2 | 0 | 0 |
| LIHC | 370 | 74 | 51 | 9 | 2 | 1 | 6 | 0 | 0 | 40 | 38 | 0 | 2 | 0 | 0 |
| PAAD | 184 | 20 | 8 | 1 | 0 | 0 | 1 | 0 | 0 | 7 | 5 | 0 | 2 | 0 | 0 |
| CHOL | 48 | 10 | 5 | 1 | 0 | 0 | 1 | 0 | 0 | 4 | 4 | 0 | 0 | 0 | 0 |
| STAD | 413 | 118 | 58 | 25 | 4 | 2 | 19 | 0 | 0 | 24 | 13 | 0 | 11 | 0 | 0 |
| OV | 583 | 107 | 58 | 18 | 11 | 2 | 3 | 0 | 2 | 30 | 23 | 0 | 5 | 1 | 1 |
| **Cancer** | **Patients** | **Num** | **PIPK** | **PIP5K1A** | | | | | | **PIP5K1B** | | | | | |
|  |  |  |  | **All** | **Amp** | **Del** | **Mutation** | **Fusion** | **Multi** | **All** | **Amp** | **Del** | **Mutation** | **Fusion** | **Multi** |
| BRCA | 1076 | 239 | 213 | 103 | 93 | 0 | 9 | 1 | 0 | 10 | 2 | 3 | 4 | 1 | 0 |
| LUAD | 509 | 84 | 69 | 40 | 34 | 0 | 5 | 0 | 1 | 4 | 0 | 1 | 3 | 0 | 0 |
| COAD | 437 | 43 | 32 | 4 | 1 | 0 | 3 | 0 | 0 | 4 | 0 | 2 | 2 | 0 | 0 |
| READ | 162 | 23 | 15 | 4 | 2 | 0 | 1 | 0 | 1 | 4 | 2 | 0 | 2 | 0 | 0 |
| GBM | 592 | 70 | 63 | 4 | 3 | 0 | 1 | 0 | 0 | 8 | 3 | 2 | 3 | 0 | 0 |
| LGG | 510 | 57 | 51 | 4 | 3 | 0 | 1 | 0 | 0 | 2 | 1 | 0 | 1 | 0 | 0 |
| LIHC | 370 | 74 | 61 | 40 | 38 | 0 | 2 | 0 | 0 | 3 | 1 | 1 | 1 | 0 | 0 |
| PAAD | 184 | 20 | 17 | 5 | 5 | 0 | 0 | 0 | 0 | 2 | 0 | 1 | 1 | 0 | 0 |
| CHOL | 48 | 10 | 9 | 4 | 4 | 0 | 0 | 0 | 0 | 0 | 0 | 0 | 0 | 0 | 0 |
| STAD | 413 | 118 | 92 | 18 | 14 | 0 | 4 | 0 | 0 | 9 | 3 | 4 | 1 | 0 | 1 |
| OV | 583 | 107 | 82 | 28 | 25 | 0 | 3 | 0 | 0 | 9 | 4 | 5 | 0 | 0 | 0 |
| **Cancer** | **Patients** | **Num** | **PIPK** | **PIP5K1C** | | | | | | **PIP4K2A** | | | | | |
|  |  |  |  | **All** | **Amp** | **Del** | **Mutation** | **Fusion** | **Multi** | **All** | **Amp** | **Del** | **Mutation** | **Fusion** | **Multi** |
| BRCA | 1076 | 239 | 213 | 14 | 4 | 4 | 4 | 2 | 0 | 10 | 8 | 0 | 2 | 0 | 0 |
| LUAD | 509 | 84 | 69 | 4 | 0 | 1 | 2 | 0 | 1 | 4 | 2 | 0 | 2 | 0 | 0 |
| COAD | 437 | 43 | 32 | 4 | 0 | 1 | 3 | 0 | 0 | 5 | 0 | 2 | 3 | 0 | 0 |
| READ | 162 | 23 | 15 | 0 | 0 | 0 | 0 | 0 | 0 | 2 | 0 | 0 | 2 | 0 | 0 |
| GBM | 592 | 70 | 63 | 8 | 3 | 1 | 4 | 0 | 0 | 4 | 1 | 1 | 2 | 0 | 0 |
| LGG | 510 | 57 | 51 | 14 | 12 | 0 | 2 | 0 | 0 | 9 | 6 | 0 | 1 | 0 | 2 |
| LIHC | 370 | 74 | 61 | 4 | 1 | 2 | 1 | 0 | 0 | 4 | 3 | 0 | 1 | 0 | 0 |
| PAAD | 184 | 20 | 17 | 2 | 1 | 0 | 1 | 0 | 0 | 1 | 0 | 0 | 1 | 0 | 0 |
| CHOL | 48 | 10 | 9 | 0 | 0 | 0 | 0 | 0 | 0 | 0 | 0 | 0 | 0 | 0 | 0 |
| STAD | 413 | 118 | 92 | 17 | 0 | 4 | 13 | 0 | 0 | 12 | 2 | 1 | 9 | 0 | 0 |
| OV | 583 | 107 | 82 | 11 | 0 | 10 | 1 | 0 | 0 | 19 | 16 | 0 | 3 | 0 | 0 |
| **Cancer** | **Patients** | **Num** | **PIPK** | **PIP4K2B** | | | | | | **PIP4K2C** | | | | | |
|  |  |  |  | **All** | **Amp** | **Del** | **Mutation** | **Fusion** | **Multi** | **All** | **Amp** | **Del** | **Mutation** | **Fusion** | **Multi** |
| BRCA | 1076 | 239 | 213 | 59 | 49 | 2 | 0 | 1 | 7 | 23 | 10 | 0 | 10 | 1 | 2 |
| LUAD | 509 | 84 | 69 | 6 | 5 | 0 | 1 | 0 | 0 | 12 | 10 | 0 | 2 | 0 | 0 |
| COAD | 437 | 43 | 32 | 8 | 6 | 0 | 2 | 0 | 0 | 3 | 0 | 0 | 3 | 0 | 0 |
| READ | 162 | 23 | 15 | 2 | 1 | 0 | 1 | 0 | 0 | 1 | 1 | 0 | 0 | 0 | 0 |
| GBM | 592 | 70 | 63 | 5 | 0 | 2 | 3 | 0 | 0 | 37 | 31 | 0 | 3 | 0 | 3 |
| LGG | 510 | 57 | 51 | 3 | 0 | 1 | 2 | 0 | 0 | 20 | 17 | 1 | 2 | 0 | 0 |
| LIHC | 370 | 74 | 61 | 6 | 3 | 2 | 1 | 0 | 0 | 3 | 2 | 0 | 1 | 0 | 0 |
| PAAD | 184 | 20 | 17 | 6 | 6 | 0 | 0 | 0 | 0 | 4 | 3 | 0 | 1 | 0 | 0 |
| CHOL | 48 | 10 | 9 | 3 | 1 | 0 | 2 | 0 | 0 | 2 | 1 | 0 | 1 | 0 | 0 |
| STAD | 413 | 118 | 92 | 22 | 18 | 0 | 4 | 0 | 0 | 10 | 7 | 0 | 3 | 0 | 0 |
| OV | 583 | 107 | 82 | 11 | 3 | 6 | 2 | 0 | 0 | 7 | 5 | 0 | 2 | 0 | 0 |
| **Cancer** | **Patients** | **Num** | **PIPK** | **PIKFYVE** | | | | | |  |  |  |  |  |  |
|  |  |  |  | **All** | **Amp** | **Del** | **Mutation** | **Fusion** | **Multi** |  |  |  |  |  |  |
| BRCA | 1076 | 239 | 213 | 27 | 7 | 5 | 14 | 0 | 1 |  |  |  |  |  |  |
| LUAD | 509 | 84 | 69 | 10 | 2 | 0 | 7 | 0 | 1 |  |  |  |  |  |  |
| COAD | 437 | 43 | 32 | 14 | 0 | 0 | 14 | 0 | 0 |  |  |  |  |  |  |
| READ | 162 | 23 | 15 | 5 | 0 | 0 | 5 | 0 | 0 |  |  |  |  |  |  |
| GBM | 592 | 70 | 63 | 8 | 0 | 0 | 8 | 0 | 0 |  |  |  |  |  |  |
| LGG | 510 | 57 | 51 | 5 | 0 | 0 | 5 | 0 | 0 |  |  |  |  |  |  |
| LIHC | 370 | 74 | 61 | 8 | 1 | 0 | 7 | 0 | 0 |  |  |  |  |  |  |
| PAAD | 184 | 20 | 17 | 4 | 4 | 0 | 0 | 0 | 0 |  |  |  |  |  |  |
| CHOL | 48 | 10 | 9 | 1 | 0 | 0 | 1 | 0 | 0 |  |  |  |  |  |  |
| STAD | 413 | 118 | 92 | 17 | 4 | 1 | 12 | 0 | 0 |  |  |  |  |  |  |
| OV | 583 | 107 | 82 | 17 | 6 | 1 | 10 | 0 | 0 |  |  |  |  |  |  |
