# Supplementary figures and images for: xinguangA preliminary characterization of PI4K/PIPK alterations across solid tumors: an exploratory framework for prognostic and therapeutic stratification
Source: Cancer Biol Ther. 2026 Jul 14;27(1):2692173. doi: 10.1080/15384047.2026.2692173 (PMC13371475; doi:10.1080/15384047.2026.2692173)

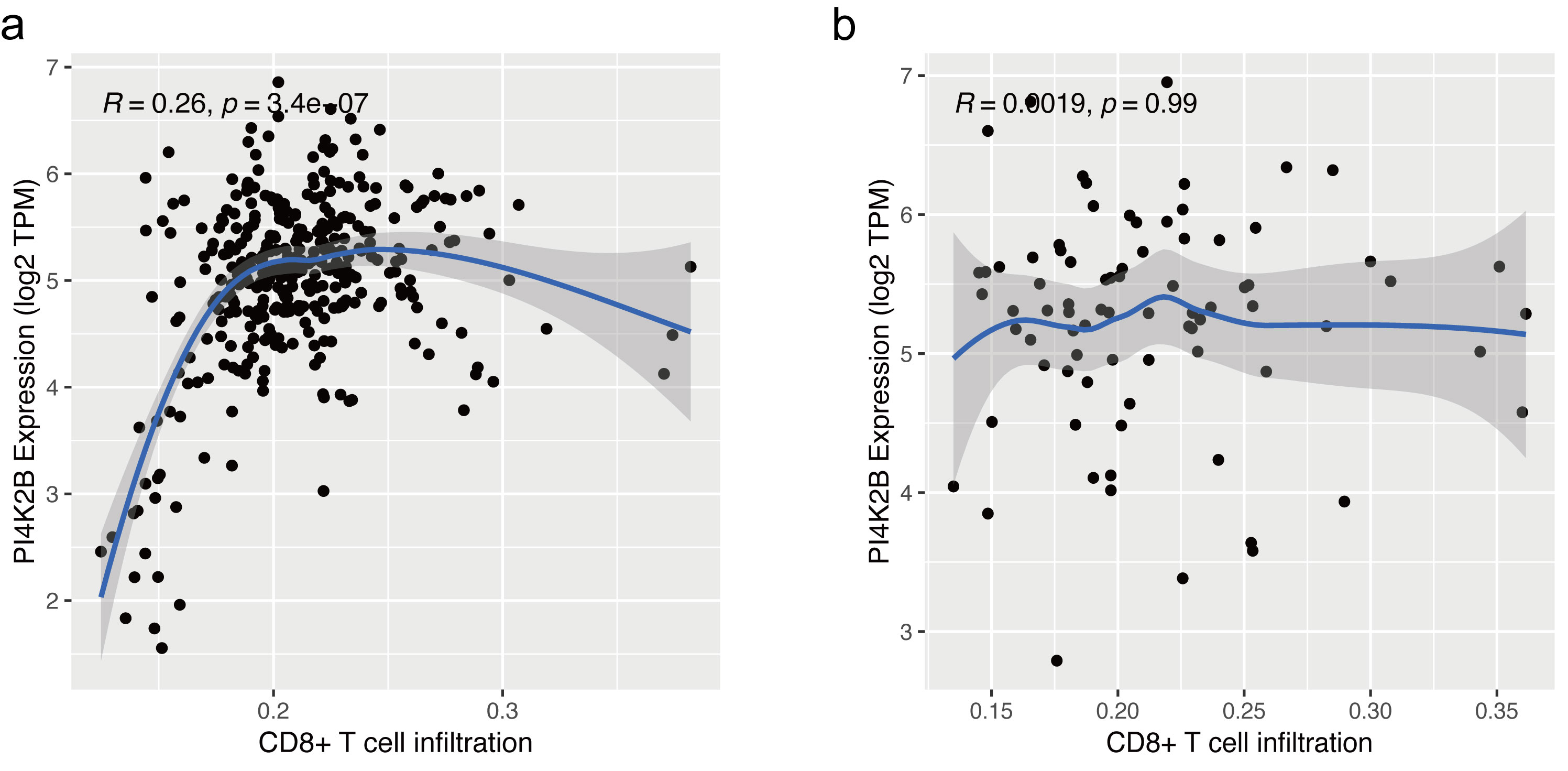

Supplement: Supplementary figure 1.jpg [file KCBT_A_2692173_SM8505.jpg]
