# Supplementary material for: xinguangA preliminary characterization of PI4K/PIPK alterations across solid tumors: an exploratory framework for prognostic and therapeutic stratification
Source: Cancer Biol Ther. 2026 Jul 14;27(1):2692173. doi: 10.1080/15384047.2026.2692173 (PMC13371475; doi:10.1080/15384047.2026.2692173)
Supplement: Supplementary Table 2.doc [file KCBT_A_2692173_SM8506.doc]

**Supplementary Table 2** Variant frequencies (%) of PI4K/PIPK in patients from the TCGA cohort

| **Cancer** | **Patients** | **Num** | **PI4K** | **PI4K2A** | | | | | | **PI4K2B** | | | | | |
| --- | --- | --- | --- | --- | --- | --- | --- | --- | --- | --- | --- | --- | --- | --- | --- |
|  |  |  |  | **All** | **Amp** | **Del** | **Mutation** | **Fusion** | **Multi** | **All** | **Amp** | **Del** | **Mutation** | **Fusion** | **Multi** |
| BRCA | 1076 | 239 | 12.27 | 0.84 | 0.09 | 0.19 | 0.28 | 0.19 | 0.09 | 0.74 | 0.19 | 0.28 | 0.28 | 0 | 0 |
| LUAD | 509 | 84 | 10.41 | 0.98 | 0 | 0.39 | 0.59 | 0 | 0 | 0.79 | 0.39 | 0 | 0.39 | 0 | 0 |
| COAD | 437 | 43 | 4.81 | 1.14 | 0 | 0.69 | 0.46 | 0 | 0 | 0.46 | 0 | 0 | 0.46 | 0 | 0 |
| READ | 162 | 23 | 8.64 | 0 | 0 | 0 | 0 | 0 | 0 | 1.23 | 0 | 0 | 1.23 | 0 | 0 |
| GBM | 592 | 70 | 2.20 | 0 | 0.17 | 0 | 0.34 | 0 | 0 | 0.68 | 0 | 0 | 0.68 | 0 | 0 |
| LGG | 510 | 57 | 2.35 | 0 | 0 | 0 | 0 | 0 | 0 | 0.39 | 0.20 | 0 | 0.20 | 0 | 0 |
| LIHC | 370 | 74 | 13.78 | 0.81 | 0.27 | 0 | 0.54 | 0 | 0 | 0.27 | 0.27 | 0 | 0 | 0 | 0 |
| PAAD | 184 | 20 | 4.35 | 0.54 | 0 | 0 | 0.54 | 0 | 0 | 0 | 0 | 0 | 0 | 0 | 0 |
| CHOL | 48 | 10 | 10.42 | 0 | 0 | 0 | 0 | 0 | 0 | 0 | 0 | 0 | 0 | 0 | 0 |
| STAD | 413 | 118 | 14.04 | 1.94 | 0 | 0.48 | 1.45 | 0 | 0 | 1.45 | 0 | 0.48 | 0.97 | 0 | 0 |
| OV | 583 | 107 | 9.95 | 0.69 | 0.34 | 0.34 | 0 | 0 | 0 | 1.37 | 0.51 | 0.17 | 0.69 | 0 | 0 |
| **Cancer** | **Patients** | **Num** | **PI4K** | **PI4KA** | | | | | | **PI4KB** | | | | | |
|  |  |  |  | **All** | **Amp** | **Del** | **Mutation** | **Fusion** | **Multi** | **All** | **Amp** | **Del** | **Mutation** | **Fusion** | **Multi** |
| BRCA | 1076 | 239 | 12.27 | 2.14 | 0.65 | 0.19 | 1.21 | 0.09 | 0 | 8.74 | 8.27 | 0 | 0.46 | 0 | 0 |
| LUAD | 509 | 84 | 10.41 | 2.36 | 0.59 | 0.20 | 1.57 | 0 | 0 | 7.47 | 6.68 | 0 | 0.59 | 0 | 0.20 |
| COAD | 437 | 43 | 4.81 | 3.20 | 0 | 0 | 3.20 | 0 | 0 | 0.69 | 0.23 | 0 | 0.46 | 0 | 0 |
| READ | 162 | 23 | 8.64 | 6.17 | 0.62 | 0 | 5.56 | 0 | 0 | 1.85 | 1.85 | 0 | 0 | 0 | 0 |
| GBM | 592 | 70 | 2.20 | 0.68 | 0.34 | 0.17 | 0.17 | 0 | 0 | 0.51 | 0.34 | 0 | 0.17 | 0 | 0 |
| LGG | 510 | 57 | 2.35 | 0.98 | 0.20 | 0 | 0.59 | 0.20 | 0 | 0.98 | 0.59 | 0 | 0.39 | 0 | 0 |
| LIHC | 370 | 74 | 13.78 | 2.43 | 0.54 | 0.27 | 1.62 | 0 | 0 | 10.81 | 10.27 | 0 | 0.54 | 0 | 0 |
| PAAD | 184 | 20 | 4.35 | 0.54 | 0 | 0 | 0.54 | 0 | 0 | 3.80 | 2.72 | 0 | 1.09 | 0 | 0 |
| CHOL | 48 | 10 | 10.42 | 2.08 | 0 | 0 | 2.08 | 0 | 0 | 8.33 | 8.33 | 0 | 0 | 0 | 0 |
| STAD | 413 | 118 | 14.04 | 6.05 | 0.97 | 0.48 | 4.60 | 0 | 0 | 5.81 | 3.15 | 0 | 2.66 | 0 | 0 |
| OV | 583 | 107 | 9.95 | 3.09 | 1.89 | 0.34 | 0.51 | 0 | 0.34 | 5.15 | 3.95 | 0 | 0.86 | 0.17 | 0.17 |
| **Cancer** | **Patients** | **Num** | **PIPK** | **PIP5K1A** | | | | | | **PIP5K1B** | | | | | |
|  |  |  |  | **All** | **Amp** | **Del** | **Mutation** | **Fusion** | **Multi** | **All** | **Amp** | **Del** | **Mutation** | **Fusion** | **Multi** |
| BRCA | 1076 | 239 | 19.80 | 9.57 | 8.64 | 0 | 0.84 | 0.09 | 0 | 0.93 | 0.19 | 0.28 | 0.37 | 0.09 | 0 |
| LUAD | 509 | 84 | 13.56 | 1.47 | 1.31 | 0 | 0.12 | 0 | 0.04 | 2.66 | 7.86 | 6.68 | 0 | 0.98 | 0 |
| COAD | 437 | 43 | 7.32 | 0.16 | 0.05 | 0 | 0.10 | 0 | 0 | 1.68 | 0.92 | 0.23 | 0 | 0.69 | 0 |
| READ | 162 | 23 | 9.26 | 1.14 | 1.14 | 0 | 0 | 0 | 0 | 5.72 | 2.47 | 1.23 | 0 | 0.62 | 0 |
| GBM | 592 | 70 | 10.64 | 0.09 | 0.06 | 0 | 0.03 | 0 | 0 | 1.80 | 0.68 | 0.51 | 0 | 0.17 | 0 |
| LGG | 510 | 57 | 10 | 0.19 | 0.12 | 0 | 0.08 | 0 | 0 | 1.96 | 0.78 | 0.59 | 0 | 0.20 | 0 |
| LIHC | 370 | 74 | 16.49 | 2.92 | 2.78 | 0 | 0.15 | 0 | 0 | 4.46 | 10.81 | 10.27 | 0 | 0.54 | 0 |
| PAAD | 184 | 20 | 9.24 | 2.07 | 1.48 | 0 | 0.59 | 0 | 0 | 5.02 | 2.72 | 2.72 | 0 | 0 | 0 |
| CHOL | 48 | 10 | 18.75 | 17.36 | 17.36 | 0 | 0 | 0 | 0 | 39.06 | 8.33 | 8.33 | 0 | 0 | 0 |
| STAD | 413 | 118 | 22.28 | 1.41 | 0.76 | 0 | 0.64 | 0 | 0 | 5.39 | 4.36 | 3.39 | 0 | 0.97 | 0 |
| OV | 583 | 107 | 14.07 | 0.88 | 0.68 | 0 | 0.15 | 0.03 | 0.03 | 2.41 | 4.80 | 4.29 | 0 | 0.51 | 0 |
| **Cancer** | **Patients** | **Num** | **PIPK** | **PIP5K1C** | | | | | | **PIP4K2A** | | | | | |
|  |  |  |  | **All** | **Amp** | **Del** | **Mutation** | **Fusion** | **Multi** | **All** | **Amp** | **Del** | **Mutation** | **Fusion** | **Multi** |
| BRCA | 1076 | 239 | 19.80 | 1.30 | 0.37 | 0.37 | 0.37 | 0.19 | 0 | 0.93 | 0.74 | 0 | 0.19 | 0 | 0 |
| LUAD | 509 | 84 | 13.56 | 0.20 | 0.79 | 0 | 0.20 | 0.59 | 0 | 0 | 0.79 | 0 | 0.20 | 0.39 | 0 |
| COAD | 437 | 43 | 7.32 | 0 | 0.92 | 0 | 0.46 | 0.46 | 0 | 0 | 0.92 | 0 | 0.23 | 0.69 | 0 |
| READ | 162 | 23 | 9.26 | 0.62 | 2.47 | 1.23 | 0 | 1.23 | 0 | 0 | 0 | 0 | 0 | 0 | 0 |
| GBM | 592 | 70 | 10.64 | 0 | 1.35 | 0.51 | 0.34 | 0.51 | 0 | 0 | 1.35 | 0.51 | 0.17 | 0.68 | 0 |
| LGG | 510 | 57 | 10 | 0 | 0.39 | 0.20 | 0 | 0.20 | 0 | 0 | 2.75 | 2.35 | 0 | 0.39 | 0 |
| LIHC | 370 | 74 | 16.49 | 0 | 0.81 | 0.27 | 0.27 | 0.27 | 0 | 0 | 1.08 | 0.27 | 0.54 | 0.27 | 0 |
| PAAD | 184 | 20 | 9.24 | 0 | 1.09 | 0 | 0.54 | 0.54 | 0 | 0 | 1.09 | 0.54 | 0 | 0.54 | 0 |
| CHOL | 48 | 10 | 18.75 | 0 | 0 | 0 | 0 | 0 | 0 | 0 | 0 | 0 | 0 | 0 | 0 |
| STAD | 413 | 118 | 22.28 | 0 | 2.18 | 0.73 | 0.97 | 0.24 | 0 | 0.24 | 4.12 | 0 | 0.97 | 3.15 | 0 |
| OV | 583 | 107 | 14.07 | 0 | 1.54 | 0.69 | 0.86 | 0 | 0 | 0 | 1.89 | 0 | 1.72 | 0.17 | 0 |
| **Cancer** | **Patients** | **Num** | **PIPK** | **PIP4K2B** | | | | | | **PIP4K2C** | | | | | |
|  |  |  |  | **All** | **Amp** | **Del** | **Mutation** | **Fusion** | **Multi** | **All** | **Amp** | **Del** | **Mutation** | **Fusion** | **Multi** |
| BRCA | 1076 | 239 | 19.80 | 5.48 | 4.55 | 0.19 | 0 | 0.09 | 0.65 | 2.14 | 0.93 | 0 | 0.93 | 0.09 | 0.19 |
| LUAD | 509 | 84 | 13.56 | 0.20 | 0.79 | 0.39 | 0 | 0.39 | 0 | 0 | 1.18 | 0.98 | 0 | 0.20 | 0 |
| COAD | 437 | 43 | 7.32 | 0 | 1.14 | 0 | 0.46 | 0.69 | 0 | 0 | 1.83 | 1.37 | 0 | 0.46 | 0 |
| READ | 162 | 23 | 9.26 | 0 | 1.23 | 0 | 0 | 1.23 | 0 | 0 | 1.23 | 0.62 | 0 | 0.62 | 0 |
| GBM | 592 | 70 | 10.64 | 0 | 0.68 | 0.17 | 0.17 | 0.34 | 0 | 0 | 0.84 | 0 | 0.34 | 0.51 | 0 |
| LGG | 510 | 57 | 10 | 0 | 1.76 | 1.18 | 0 | 0.20 | 0 | 0.39 | 0.59 | 0 | 0.20 | 0.39 | 0 |
| LIHC | 370 | 74 | 16.49 | 0 | 1.08 | 0.81 | 0 | 0.27 | 0 | 0 | 1.62 | 0.81 | 0.54 | 0.27 | 0 |
| PAAD | 184 | 20 | 9.24 | 0 | 0.54 | 0 | 0 | 0.54 | 0 | 0 | 3.26 | 3.26 | 0 | 0 | 0 |
| CHOL | 48 | 10 | 18.75 | 0 | 0 | 0 | 0 | 0 | 0 | 0 | 6.25 | 2.08 | 0 | 4.17 | 0 |
| STAD | 413 | 118 | 22.28 | 0 | 2.91 | 0.48 | 0.24 | 2.18 | 0 | 0 | 5.33 | 4.36 | 0 | 0.97 | 0 |
| OV | 583 | 107 | 14.07 | 0 | 3.26 | 2.74 | 0 | 0.51 | 0 | 0 | 1.89 | 0.51 | 1.03 | 0.34 | 0 |
| **Cancer** | **Patients** | **Num** | **PIPK** | **PIKFYVE** | | | | | |  |  |  |  |  |  |
|  |  |  |  | **All** | **Amp** | **Del** | **Mutation** | **Fusion** | **Multi** |  |  |  |  |  |  |
| BRCA | 1076 | 239 | 19.80 | 2.51 | 0.65 | 0.46 | 1.30 | 0 | 0.09 |  |  |  |  |  |  |
| LUAD | 509 | 84 | 13.56 | 0 | 2.36 | 1.96 | 0 | 0.39 | 0 |  |  |  |  |  |  |
| COAD | 437 | 43 | 7.32 | 0 | 0.69 | 0 | 0 | 0.69 | 0 |  |  |  |  |  |  |
| READ | 162 | 23 | 9.26 | 0 | 0.62 | 0.62 | 0 | 0 | 0 |  |  |  |  |  |  |
| GBM | 592 | 70 | 10.64 | 0 | 6.25 | 5.24 | 0 | 0.51 | 0 |  |  |  |  |  |  |
| LGG | 510 | 57 | 10 | 0 | 3.92 | 3.33 | 0.20 | 0.39 | 0 |  |  |  |  |  |  |
| LIHC | 370 | 74 | 16.49 | 0 | 0.81 | 0.54 | 0 | 0.27 | 0 |  |  |  |  |  |  |
| PAAD | 184 | 20 | 9.24 | 0 | 2.17 | 1.63 | 0 | 0.54 | 0 |  |  |  |  |  |  |
| CHOL | 48 | 10 | 18.75 | 0 | 4.17 | 2.08 | 0 | 2.08 | 0 |  |  |  |  |  |  |
| STAD | 413 | 118 | 22.28 | 0 | 2.42 | 1.69 | 0 | 0.73 | 0 |  |  |  |  |  |  |
| OV | 583 | 107 | 14.07 | 0 | 1.20 | 0.86 | 0 | 0.34 | 0 |  |  |  |  |  |  |
