# Supplementary material for: xinguangA preliminary characterization of PI4K/PIPK alterations across solid tumors: an exploratory framework for prognostic and therapeutic stratification
Source: Cancer Biol Ther. 2026 Jul 14;27(1):2692173. doi: 10.1080/15384047.2026.2692173 (PMC13371475; doi:10.1080/15384047.2026.2692173)
Supplement: Supplementary Table 4.doc [file KCBT_A_2692173_SM8507.doc]

**Supplementary Table 4** Variants frequencies (%) of PI4K/PIPK genes by race in the TCGA cohort

| **cancer** | **PI4K** | | | **PI4K2A** | | | **PI4K2B** | | | **PI4KA** | | | **PI4KB** | | |
| --- | --- | --- | --- | --- | --- | --- | --- | --- | --- | --- | --- | --- | --- | --- | --- |
|  | **asian** | **non-asian** | **p** | **asian** | **non-asian** | **p** | **asian** | **non-asian** | **p** | **asian** | **non-asian** | **p** | **asian** | **non-asian** | **p** |
| BRCA | 4.92 | 2.66 | 0.24 | 1.64 | 0.21 | 0.172 | 0 | 0.32 | 1 | 1.64 | 1.70 | 1 | 1.64 | 1.06 | 0.501 |
| CHOL | 0 | 0 | 1 | 0 | 0 | 1 | 0 | 0 | 1 | 0 | 0 | 1 | 0 | 0 | 1 |
| COAD | 27.27 | 10.58 | 0.114 | 18.18 | 0.73 | 0.008 | 9.09 | 1.82 | 0.212 | 9.09 | 5.84 | 0.498 | 9.09 | 4.38 | 0.407 |
| READ | 0 | 7.95 | 1 | 0 | 0 | 1 | 0 | 1.14 | 1 | 0 | 6.82 | 1 | 0 | 0 | 1 |
| GBM | 0 | 2.51 | 1 | 0 | 0.54 | 1 | 0 | 0.54 | 1 | 0 | 1.43 | 1 | 0 | 0.90 | 1 |
| LGG | 0 | 1.41 | 1 | 0 | 0 | 1 | 0 | 0.20 | 1 | 0 | 1.01 | 1 | 0 | 0.60 | 1 |
| LIHC | 2.48 | 3.40 | 0.762 | 0 | 0.97 | 0.506 | 0 | 0 | 1 | 1.86 | 2.43 | 1 | 1.24 | 0 | 0.192 |
| LUAD | 0 | 6.95 | 1 | 0 | 0.45 | 1 | 0 | 0.22 | 1 | 0 | 3.59 | 1 | 0 | 2.91 | 1 |
| OV | 0 | 5.05 | 0.616 | 0 | 0.19 | 1 | 0 | 0.93 | 1 | 0 | 2.06 | 1 | 0 | 2.24 | 1 |
| PAAD | 0 | 1.18 | 1 | 0 | 1.18 | 1 | 0 | 0 | 1 | 0 | 0.59 | 1 | 0 | 0.59 | 1 |
| STAD | 0 | 8.90 | 0.001 | 0 | 1.37 | 0.577 | 0 | 1.71 | 0.595 | 0 | 3.42 | 0.125 | 0 | 5.14 | 0.027 |
| **cancer** | **PIPK** | | | **PIP5K1A** | | | **PIP5K1B** | | | **PIP5K1C** | | | **PIP4K2A** | | |
|  | **asian** | **non-asian** | **p** | **asian** | **non-asian** | **p** | **asian** | **non-asian** | **p** | **asian** | **non-asian** | **p** | **asian** | **non-asian** | **p** |
| BRCA | 1.64 | 4.14 | 0.507 | 1.64 | 1.06 | 0.501 | 0 | 0.43 | 1 | 0 | 0.32 | 1 | 0 | 0.32 | 1 |
| CHOL | 0 | 8.11 | 1 | 0 | 2.70 | 1 | 0 | 0 | 1 | 0 | 0 | 1 | 0 | 0 | 1 |
| COAD | 27.27 | 13.50 | 0.189 | 9.09 | 0.36 | 0.076 | 0 | 1.46 | 1 | 9.09 | 4.38 | 0.407 | 0 | 3.65 | 1 |
| READ | 0 | 7.95 | 1 | 0 | 1.14 | 1 | 0 | 2.27 | 1 | 0 | 0 | 1 | 0 | 3.41 | 1 |
| GBM | 0 | 5.91 | 1 | 0 | 0.90 | 1 | 0 | 0.90 | 1 | 0 | 0.72 | 1 | 0 | 0.90 | 1 |
| LGG | 0 | 1.81 | 1 | 0 | 0.20 | 1 | 0 | 0.20 | 1 | 0 | 0.20 | 1 | 0 | 0.20 | 1 |
| LIHC | 5.59 | 4.85 | 0.815 | 0.62 | 0.49 | 1 | 1.24 | 0 | 0.192 | 0 | 0.49 | 1 | 0 | 0.49 | 1 |
| LUAD | 0 | 8.52 | 1 | 0 | 0.67 | 1 | 0 | 1.57 | 1 | 0 | 0.45 | 1 | 0 | 0.90 | 1 |
| OV | 5.00 | 6.54 | 1 | 5.00 | 0.93 | 0.198 | 0 | 0.37 | 1 | 0 | 0.37 | 1 | 0 | 0.56 | 1 |
| PAAD | 9.09 | 0.59 | 0.119 | 0 | 0.59 | 1 | 0 | 0.59 | 1 | 0 | 0.59 | 1 | 9.09 | 0 | 0.061 |
| STAD | 13.48 | 9.59 | 0.324 | 1.12 | 0.68 | 0.551 | 0 | 0.34 | 1 | 4.49 | 3.42 | 0.747 | 0 | 2.40 | 0.207 |
| **PIP4K2B** | | | **PIP4K2C** | | | **PIKFYVE** | | |  |  |  |  |  |  |  |
| **asian** | **non-asian** | **p** | **asian** | **non-asian** | **p** | **asian** | **non-asian** | **p** |  |  |  |  |  |  |  |
| 0 | 0.21 | 1 | 0 | 1.17 | 1 | 0 | 1.91 | 0.62 |  |  |  |  |  |  |  |
| 0 | 0 | 1 | 0 | 0 | 1 | 0 | 5.41 | 1 |  |  |  |  |  |  |  |
| 0 | 1.82 | 1 | 0 | 0.36 | 1 | 9.09 | 13.50 | 1 |  |  |  |  |  |  |  |
| 0 | 2.27 | 1 | 0 | 0 | 1 | 0 | 2.27 | 1 |  |  |  |  |  |  |  |
| 0 | 1.25 | 1 | 0 | 0.54 | 1 | 0 | 2.69 | 1 |  |  |  |  |  |  |  |
| 0 | 0.20 | 1 | 0 | 0.40 | 1 | 0 | 0.80 | 1 |  |  |  |  |  |  |  |
| 0 | 0.49 | 1 | 0.62 | 0.49 | 1 | 3.11 | 2.43 | 0.754 |  |  |  |  |  |  |  |
| 0 | 0.45 | 1 | 0 | 1.79 | 1 | 0 | 3.81 | 1 |  |  |  |  |  |  |  |
| 0 | 0.75 | 1 | 0 | 0.37 | 1 | 0 | 3.55 | 1 |  |  |  |  |  |  |  |
| 0 | 0.59 | 1 | 0 | 0.59 | 1 | 0 | 0 | 1 |  |  |  |  |  |  |  |
| 3.37 | 1.37 | 0.361 | 2.25 | 0.34 | 0.138 | 3.37 | 5.14 | 0.775 |  |  |  |  |  |  |  |
