# Supplementary material for: xinguangA preliminary characterization of PI4K/PIPK alterations across solid tumors: an exploratory framework for prognostic and therapeutic stratification
Source: Cancer Biol Ther. 2026 Jul 14;27(1):2692173. doi: 10.1080/15384047.2026.2692173 (PMC13371475; doi:10.1080/15384047.2026.2692173)
Supplement: Supplementary Material [file KCBT_A_2692173_SM4890.docx]

Supplementary Figure 1. Stratified correlation analysis between PI4K2B expression and CD8+ T cell infiltration by microsatellite status.
